# Anorectal avulsion: an exceptional rectal trauma

**DOI:** 10.1186/1749-7922-8-40

**Published:** 2013-10-07

**Authors:** Karim Ibn majdoub Hassani, Said Ait laalim, El Bachir Benjelloun, Imane Toughrai, Khalid Mazaz

**Affiliations:** 1 Department of Surgery, School of Medicine and Pharmacy of Fez, Sidi Mohammed Ben Abdellah University, BP: 1893; Km2.200, Route de Sidi Hrazem, Fez 30000, Morocco

**Keywords:** Anorectal avulsion, Rectal trauma, Surgical management

## Abstract

Anorectal avulsion is an exceptional rectal trauma in which the anus and sphincter no longer join the perineum and are pulled upward. As a result, they ventrally follow levator ani muscles. We present a rare case of a 29-years old patient who was admitted in a pelvic trauma context; presenting a complete complex anorectal avulsion. The treatment included a primary repair of the rectum and a diverting colostomy so as to prevent sepsis. Closure of the protective sigmoidostomy was performed seven months after the accident and the evolution was marked by an anal stenosis requiring iterative dilatations.

## Introduction

Anorectal avulsion is an exceptional rectal trauma. In this kind of lesions, the anus and sphincter no longer join the perineum and are pulled upward. They are in addition ventrally following levator ani muscles. The management of this kind of lesions remains a matter of great debate. Early repair of the rectum, diverting colostomy, wound debridement, distal rectal wash-out are the most important procedures that help prevent sepsis. In addition, the colostomy closure can only be performed after pelvic rehabilitation in order to prevent transitory incontinence.

## Observation

**Figure 1:**
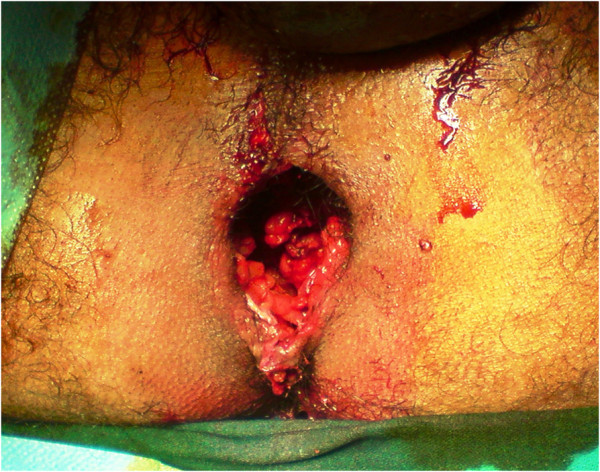
Inspection of the perineum showing a big loss of substance with complete avulsion of anorectal complex.

**Figure 2:**
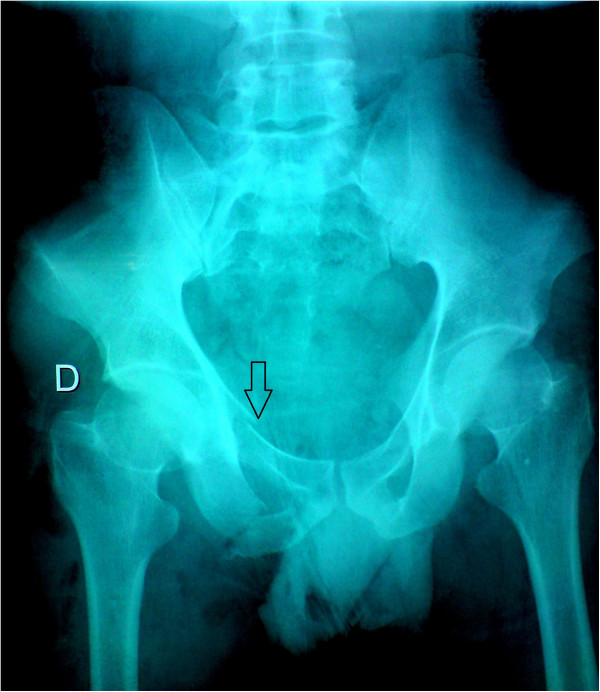
Pelvic X-ray showing a right ischio pubic rami fracture.

**Figure 3:**
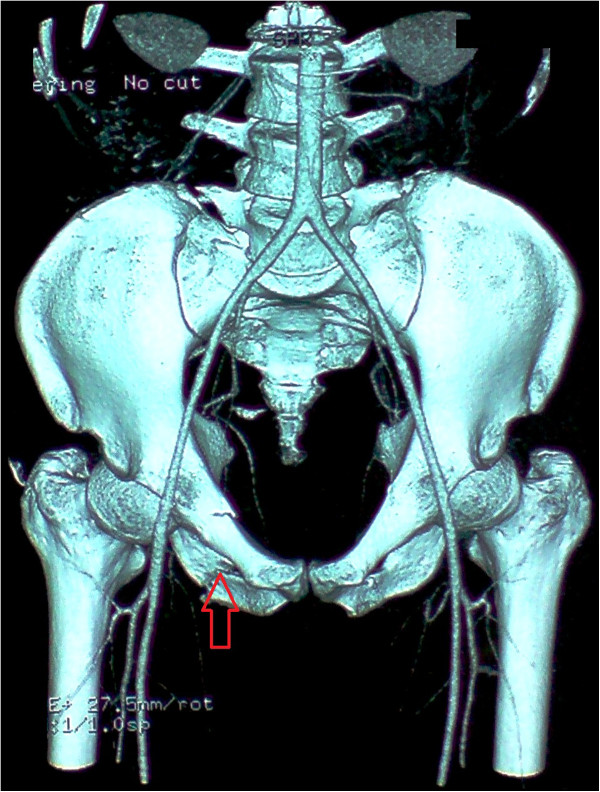
Computed tomography (CT) showing a right ischio pubic rami fracture.

**Figure 4:**
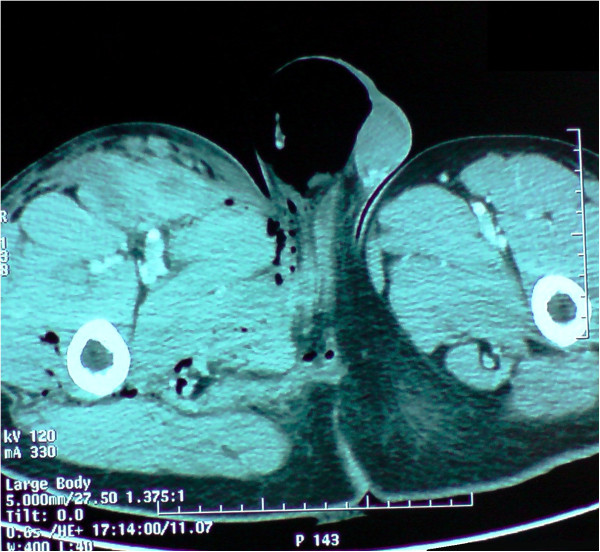
CT showing a right scrotal Pneumatocele.

**Figure 5:**
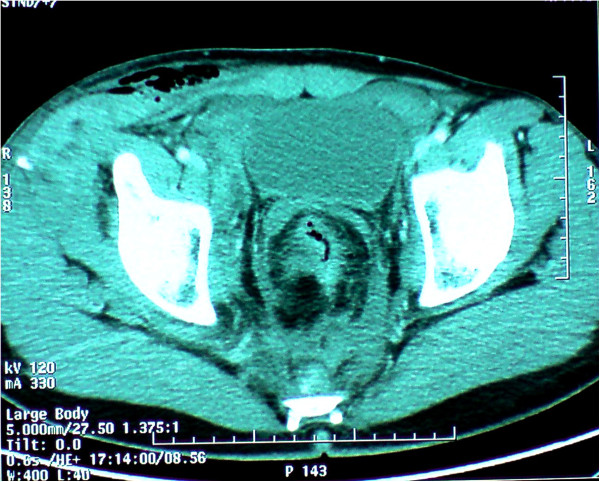
CT showing free air in perirectal space and in the right lateral abdominal wall.

**Figure 6:**
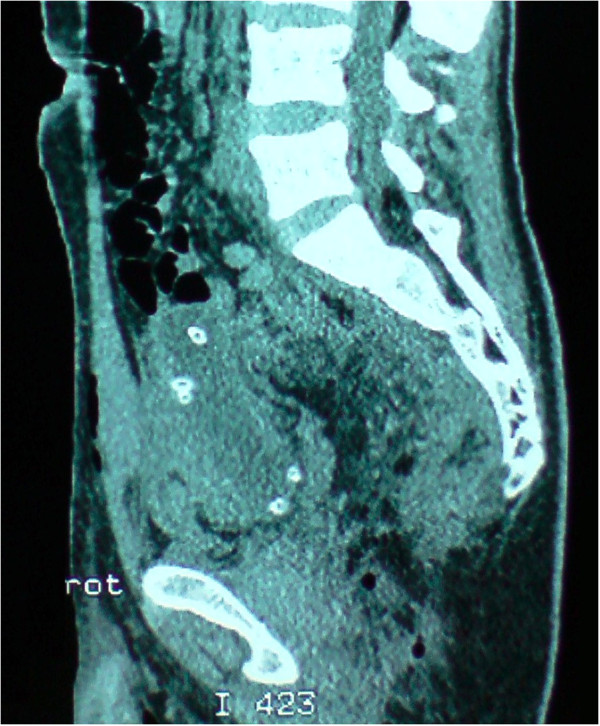
Coronal coupe showing the anorectal avulsion with free air in the perirectal space.

**Figure 7:**
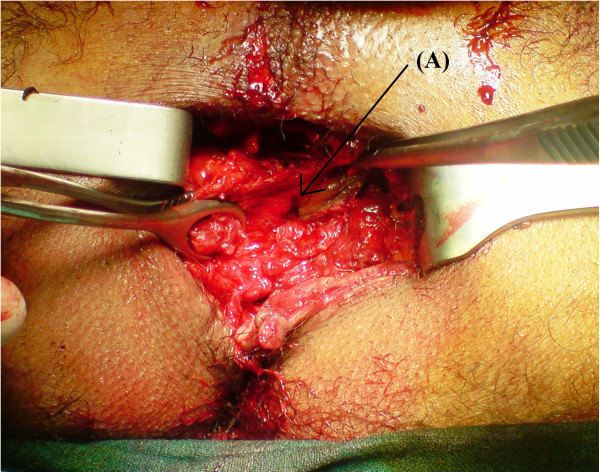
The perineum examination showing anorectal stump retracted upward and ventrally (A: rectal lumen).

**Figure 8:**
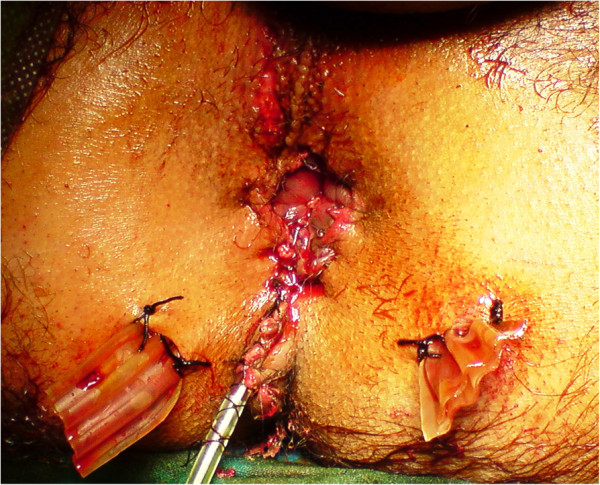
Perineal skin closed with presacral drainage.

**Figure 9:**
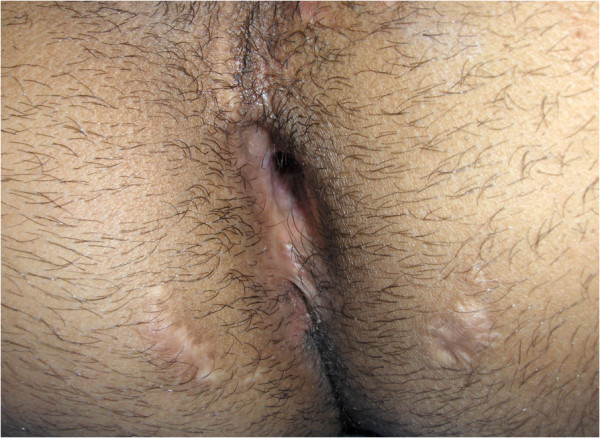
Final aspect of the anal margin six months after anorectal repair.

A 29-years-old patient was admitted to the emergency room (ER) of the University hospital Hassan II of Fez after having an accident which resulted in a severe pelvic trauma. When the patient was admitted to the ER, he was agitated but conscious and hemodynamically stable with slightly discolored conjunctives. The physical examination revealed a pulse rate of 90 beat per minute, a blood pressure of 110/80 mmHg, but there was no fever. Abdominal examination showed minimal tenderness in the hypogastria with a distended bladder. Urologic examination revealed urethral bleeding with a large scrotal scar. The perineal exam showed a big substance loss with complete anorectal avulsion due to the contraction of the elevator ani muscle (Figure 1). Laboratory data showed a white-blood cell count of 10 900/mm3, serum hemoglobin concentration of 10,4 g/dl with a normal blood platelet level (390,000/mm3), a blood urea of 0.45 g/l and a creatinine level of 10 mg/L. Hemostasis laboratory data, chemistry and serum lipase were within normal limits. So, being hemodynamic stable, the patient underwent chest X-ray. The latter was normal. The pelvic X-ray showed a right ischio pubic rami fracture (Figure 2). A contrast-enhanced computed tomography (CT) was performed and therefore showed a pelvic trauma with right ischio pubic rami fracture (Figure 3) as well as a fracture in the right transverse process of L5 and S1 sacral wing. CT scan also showed a right bladder effusion extending to the retro peritoneal area. Furthermore, there was a large inguinal hematoma measuring 10 x 4 cm and fusing along the right thigh. It was therefore associated with symphysis emphysematous soft tissue extending down to the scrotum the thing that resulted in a right scrotal pneumatocele (Figure 4). There was also free air in the perineum, the perirectal space and the right lateral abdominal wal (Figures 5, 6). No free abdominal fluid or air was detected. The patient was taken to the operating room. Suprapubic cyst catheter was placed. During the perineal exam, the anorectal stump was hardly recognized among the injured tissues for it was retracted upward and ventrally making the distance between the anal canal and the perineal skin about 6 cm (Figure 7). A rectal washout was performed. Necrosectomy with several debridements as well as presacral irrigation were realized. The ano-rectal mucosa was closed at first; then the torn ends of the external sphincter were identified and sutured accurately. Presacral drainage was placed in the ischio rectal area by a passive drain and delbet lames (Figure 8). Finally the perineal skin was closed using good mattress sutures to build up the perineal body. A sigmoid loop colostomy was performed through an elective laparotomy in the left iliac fossa. As far as the treatment is concerned, the patient was given an antibiotic regimen consisting of ciprofloxacin and metronidazole for two weeks. The postoperative course was unremarkable. Drainage was removed at the fifth day after surgery. Conservative treatment was undertaken for spine and rib fracture. Anorectal Manometry was performed six months after surgery. The latter did not show any physiologic dysfunction except the length of the anal canal which was reduced to less than 2 cm (Figure 9). Sigmoidostomy closure was performed seven months after the surgery. Unfortunately, the evolution was marked by anal stenosis which required iterative dilatations. Nowadays, during 9 months of follow up, the patient is free of any symptoms since the very last dilatation.

## Discussion

Although there are similarities between colonic injuries and rectal ones, there are also differences which are unique to the rectum. Approximately 80% of rectal injuries are attributable to firearms and less than 3% are secondary to stab or impalement etiologies. Less than 10% of rectal injuries are blunt by nature as a result of falls, motor vehicle accidents or pelvic fractures [1]. While the management of rectal injuries has changed over the last few years, optimal treatment remains a matter of great debate. The anorectal avulsion is a particular case of rectal injuries. It’s a very rare rectal trauma. After reviewing the literature, we found out that the first case of post traumatic anorectal avulsion was reported in 1965 by Mathieson et al. [2]. During the following years, only few case reports were described (Table 1) [3-6]. In this kind of lesions, the anus and sphincter no longer join the perineum and are pulled upward and thus ventrally follow levator ani muscles. In addition, their treatment is controversial and not standardized [7]. A multidisciplinary approach is mandatory involving general surgeons, anesthetists and rehabilitators [8,9]. The main difficulties encountered when treating these lesions are: to prevent sepsis and keep good anal sphincter functions at the same time. Management strategies described in the literature include diverting sigmoidostomy, presacral drainage, direct suture repair of the rectal laceration and irrigation of the rectum. In 1989, Burch et al. [10] recommended fecal diversion and presacral drainage for rectal injury management. The primary repair of a rectal lesion should be always tried if local conditions allow it. This was the case of our patient in which direct suture was difficult to perform but was still possible. Presacral drainage is believed to prevent perirectal infections due to fecal contamination and has been used widely to reduce abscess formation in extraperitoneal rectal trauma. This evidence derives mainly by war injury [7], but some authors [9,11,12] demonstrated no difference in infection rates associated with civilian rectal trauma caused by low velocity injury. Diverting colostomy has been demonstrated safe and effective in reducing the infection rate associated with rectal trauma 8 and a valid tool to perform rectal wash-out. However, in a study by Gonzales [13], fourteen patients suffering from non-destructive penetrating extraperitoneal rectal injuries were treated without fecal diversion or direct suture repair. Infectious complications didn’t occur in any of these patients. Furthermore, Navsaria and colleagues concluded from their retrospective review that extraperitoneal rectal injuries caused by low-velocity penetrating trauma could be treated only by fecal diversion [9]. Although there are controversies concerning the colostomy type, the drainage method, the need for distal washout, and the need to repair the rectal wound, most trauma surgeons as it is the case with our surgical tream, would agree about the need for diversion and drainage in the management of extraperitoneal rectal injuries in addition to primary repair of rectal lesion which should always be tried if local conditions allow it [14,15].

**Table 1:** Reported cases of anorectal avulsion

**Authors**	**Year**	**Title**	**Management of the anorectal avulsion**
Mathieson, A. J et al.	1965	Rupture of the posterior urethra and avulsion of the rectum and anus as a complication of fracture of the pelvis	Primary repair + presacral drainage + sigmoid loop colostomy
Sharma D. et al	2000	Anorectal avulsion: an unusual rectal injury	Primary repair + presacral drainage + sigmoid loop colostomy
Terrosu G. et al	2011	Anal avulsion caused by abdominal crush injury	Anal reimplantation + pelvic drainage tubes + loop transverse colostomy
Rispoli C. et al.	2012	Anorectal avulsion: Management of a rare rectal trauma	Direct suture not possible sigmoid loop colostomy + presacral drainage + anoperineal reparation 10 weeks later
R. M. Gomesa et al	2013	Anorectal avulsion: report of a rare case of rectal injury	diverting sigmoid loop colostomy (primary repair not possible)

## Consent

Written informed consent was obtained from the patient for publication of this Case report and any accompanying images.

## Competing interests

All authors declare no competing interests.

## Authors’ contributions

KIM and SA participated in writing the case report and revising the draft, IT took the photos E B and KM participated in the follow up. All authors read and approved the final manuscript.

## Authors’ information

School of Medicine And Pharmacy of Fez, Sidi Mohammed Ben Abdellah University Department of Surgery, University hospital HASSAN II, BP: 1893; Km2.200, Route de Sidi Hrazem; FEZ 30000, MOROCCO.

## References

[B1] Cintron JR Colon and rectum traumahttp://www.fascrs.org/physicians/education/core_subjects/2006/colon_rectal_trauma/

[B2] Mathieson AJM Mann TS Rupture of the posterior urethra and avulsion of the rectum and anus as a complication of fracture of the pelvis Brit J Surg 1965 8 309 10.1002/bjs.1800520416 14271095

[B3] Sharma D Rahman H Mandloi KC Saxena A Raina VK Kapoor JP Anorectal avulsion: an unusual rectal injury Digestive Surg 2000 8 193 194 PubMed: 10781991 10.1159/000018831 10781991

[B4] Terrosu G Rossetto A Kocjancic E Rossitti P Bresadola V Anal avulsion caused by abdominal crush injury Tech in Coloproctology 2011 8 465 468 [PubMed: 21556880] 10.1007/s10151-011-0680-x 21556880

[B5] Rispoli C Andreuccetti J Iannone L Anorectal avulsion: management of a rare rectal trauma Int J Surg Case Rep 2012 8 319 321 10.1016/j.ijscr.2012.04.001 22554940 PMC3356549

[B6] Gomesa RM Kudchadkara J Araujob E Gundawarc T Anorectal avulsion: report of a rare case of rectal injury, letter to the editor Ann Gastroenterology 2013 8 1 PMC3959477 24714283

[B7] Velmahos GC Gomez H Falabella A Demetriades D Operative management of civilian rectal gunshot wounds: simpler is better World J Surg 2000 8 1 114 118 PubMed: 10594214 10.1007/s002689910021 10594214

[B8] Cleary RK Pomerantz RA Lampman RM Colon and rectal injuries Dis Colon and Rectum 2006 8 8 1203 1222 PubMed: 16858663 10.1007/s10350-006-0620-y 16858663

[B9] Navsaria PH Edu S Nicol AJ Civilian extraperitoneal rectal gunshot wounds: surgical management made simpler World J Surg 2007 8 6 1345 1351 PubMed: 17457641 10.1007/s00268-007-9027-1 17457641

[B10] Burch MD JM Feliciano MD DV Mattox MD KL Colostomy and drainage for civilian rectal injuries: is that all? Ann Surg 1989 8 5 600 610 discussion 610-1 10.1097/00000658-198905000-00013 2705824 PMC1494065

[B11] Gonzalez RP Falimirski ME Holevar MR The role of presacral drainage in the management of penetrating rectal injuries J Trauma 1998 8 4 656 661 PubMed: 9783600 10.1097/00005373-199810000-00002 9783600

[B12] Armstrong RG Schmitt HJ Jr Patterson LT Combat wounds of the extraperitoneal rectum Surgery 1973 8 570 574 PubMed: 4729222 4729222

[B13] Gonzalez RP Phelan H 3rd Hassan M Ellis CN Rodning CB Is fecal diversion necessary for nondestructive penetrating extraperitoneal rectal injuries ? J Trauma 2006 8 4 815 819 10.1097/01.ta.0000239497.96387.9d 17033545

[B14] Burch JM Feliciano DV Mattox KL Colostomy and drainage for civilian rectal injuries: is that all? Ann Surg 1989 8 5 600 610 10.1097/00000658-198905000-00013 2705824 PMC1494065

[B15] Ivatury RR Licata J Gunduz Y Rao P Stahl WM Management options in penetrating rectal injuries Am Surg 1991 8 1 50 55 1796798

